# An interplay of microglia and matrix metalloproteinase MMP9 under hypoxic stress regulates the opticin expression in retina

**DOI:** 10.1038/s41598-021-86302-2

**Published:** 2021-04-02

**Authors:** Satish Patnaik, Meenakshi Rai, Subhadra Jalali, Komal Agarwal, Akshay Badakere, Lavanya Puppala, Sushma Vishwakarma, Divya Balakrishnan, Padmaja K. Rani, Ramesh Kekunnaya, Preeti Patil Chhablani, Subhabrata Chakrabarti, Inderjeet Kaur

**Affiliations:** 1 Prof. Brien Holden Eye Research Centre, LV Prasad Eye Institute, Hyderabad, India; 2 Department of Animal Biology, School of Life Sciences, University of Hyderabad, Hyderabad, Telangana India; 3 Smt. Kannuri Santhamma Centre for Vitreo Retinal Diseases, Hyderabad, India; 4 Jasti V Ramanamma Children’s Eye Care Centre, LV Prasad Eye Institute, Hyderabad, India; 5 University of Pittsburgh Medical Center, Pittsburgh, USA

**Keywords:** Biochemistry, Biological techniques, Drug discovery, Molecular biology, Biomarkers, Diseases, Pathogenesis, Risk factors

## Abstract

Inflammation plays a key role in the pathogenesis of retinal vascular diseases. We have shown earlier an increase in the activity of matrix metalloproteinases in the vitreous and tears of preterm born babies with retinopathy of prematurity (ROP) compared to those with no-ROP leading to a shift in the balance of angiogenic (vascular endothelial growth factor [VEGF], matrix metalloproteinase [MMPs], complement component [C3]) and anti-angiogenic (opticin, thrombospondin) in ROP eyes. We now confirmed that tear MMP levels in premature infants perfectly correlates with disease severity. Next, we demonstrated that a reduced opticin levels in ROP vitreous are regulated by MMPs secreted by activated microglia. Upon exposing the human microglia cell line (CHME3) to hypoxia, an increased expression of inflammatory proteins (MMP9, VEGF) was noticed while opticin reduced significantly (*p* = 0.005). Further, the reduced opticin’s expression by microglial cells under hypoxia could be rescued by inhibiting the MMP activity using doxycycline and EDTA. The inhibition of MMP activity altered the expression of other key signaling molecules under hypoxia. Our study clearly explains that increased activity of MMPs under hypoxia regulates the expression of opticin as seen in the vitreous humor of ROP and could serve as a potential target for ROP management.

## Introduction

Retinopathy of prematurity (ROP) is one of the most common causes of childhood blindness^1^. It is a vaso-proliferative eye disease in premature babies characterized by abnormal blood vessel growth in the retina that can cause retinal detachment and eventually lead to blindness^2^. Annually in India, nearly 1.2 million babies are prone to develop ROP^3^. The reported risk factors for ROP include low birth weight, low gestational age, gender, ethnicity, light exposure, blood transfusion and other maternal risk factors. However, gestational age, birth-weight and oxygen supplementation (to some extent) have been found to be the key defining factors across multiple studies worldwide^4^. ROP is an exceedingly complex disease, as in some set of premature infants, it regresses spontaneously while in others, it progresses from mild form (ROP-stage I, II) to severe form (ROP-stage III, IV,V) eventually leading to total vision loss if not treated timely. While ROP is considered to be a hypoxia driven neovascular condition, till date the underlying molecular mechanisms contributing to pathogenesis of ROP remain unclear. Probably, various pathological disease-causing risk factors together lead to the progression of ROP. Most commonly proposed mechanism for the pathogenesis of ROP are hypoxia induced vaso-attenuation and vaso-proliferation leading to oxidative stress^5^ that further induces the release of pro-inflammatory and pro-angiogenic molecules in ROP patients^6^. All these factors together play a crucial role in the progression of the disease.

ROP being a vitreoretinal condition, alters the homeostatic balance of anti- and angiogenic proteins in the vitreous^6^. Vitreous gel is optically transparent and is composed of diverse proteins (collagens, albumin, IgG, oxidative stress enzymes and cytokines), proteoglycans (hyaluronan and chondroitin sulfate proteoglycans) and small molecules^7^. Due to proximity of vitreous with retina, especially when the blood-retinal barrier (BRB) is damaged, the secretory product of retina gets accumulated in the vitreous^8^. Moreover, in vitreoretinal diseases, the vitreous composition also changes due to differential expression of proteins under various disease conditions^7^. Therefore, studying differential protein profiling in the vitreous gel may help in understanding the underlying mechanisms of ROP.

Immunohistochemistry of the ridge membrane formed at stage II showed by the presence of endothelial cells, macroglial cells, microglia and few proliferating cells^9^. However, the exact role of microglia in ROP pathogenesis is not completely understood. Findings from our recent study on ROP suggested that activated microglia secretes elevated levels of matrix metalloproteinase (MMPs) and pro-inflammatory molecules into the vitreous and the retina^10^. The elevated levels of activated MMPs might cause extracellular matrix (ECM) degradation, in turn, promoting angiogenesis. The significant increase in MMP9, tissue inhibitory metalloproteases (TIMP1), and α2 macroglobulin in the ROP vitreous further confirmed it. Dysregulation of MMP2, and MMP9 were also detected reproducibly in stage dependent manner in ROP tear samples^10^.

A pilot vitreous proteome profiling study (unpublished data) by our group revealed a lower expression of opticin, an anti-angiogenic protein in ROP patients. Opticin is present abundantly in human vitreous, and secreted into the vitreous cavity by non-pigmented epithelial cells constantly to maintain its balanced levels^11^. Opticin is an extra cellular matrix (ECM) glycoprotein associated with the collagen fibrils and the retinal growth hormone^12^ in the vitreous cavity. It is also identified in the other tissues like cartilage, brain and heart^11,13^. The 332 amino acid protein has a sequence that is homologous to class III small leucine rich proteins (SLRPs), epiphycan and osteoglycan, with a consensus sequence of LXXLXLXXNXL. These SLRP’s confined by conserved cysteine residues interact with ECM. The role of opticin in angiogenesis has been previously identified in murine oxygen mouse model and cell culture model^14,15^. The opticin knockout model showed increased neovascularization compared to wild type mice^14^. Based on this background, we hypothesized that the abnormal activity of MMPs under hypoxic conditions affects the opticin levels and thereby contributes to increased inflammation in the retina causing ROP progression.

## Results

### Role of ECM proteins in ROP

**Figure 1:**
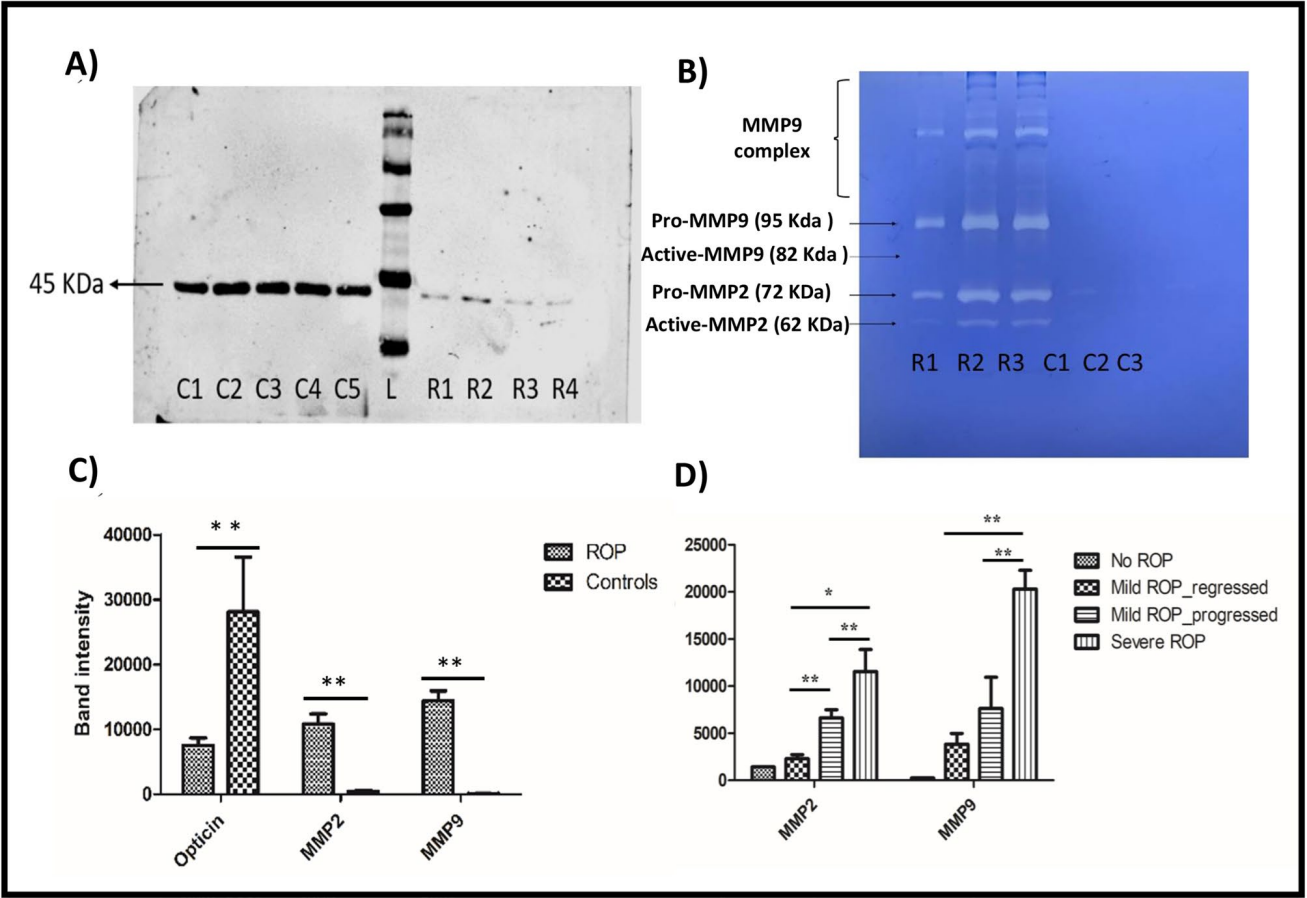
Representative image of (**A**) western blot of opticin levels in ROP patients and controls (**B**) representative gelatin zymography of vitreous from ROP and congenital cataract (controls) (**C**) quantification of opticin (45 K Da) levels in vitreous samples of ROP (n = 30) and control (n = 30) and MMPs in ROP (n = 11) and control (n = 11) (**D**) MMPs levels estimated in ROP tears samples by zymography in extended cohort, quantification of MMP9 and MMP2 estimated for severe ROP (n = 16), mild progressed to progressed ROP (n = 12), mild ROP to regressed ROP (n = 12), and no ROP premature controls (n = 18), **p = 0.001, *p = 0.05; data represented as mean ± SEM, C, control vitreous; R, ROP vitreous; L, protein ladder.

The western blotting and zymography for ECM proteins including opticin and MMPs (MMP2, and MMP9) respectively showed a downregulation of opticin (45 k Da) with an increase in MMPs activity in the vitreous samples of ROP patients (Fig. 1A and B; Supplementary Fig. 1). The differential expression of MMP2 (*p*-value = 0.00001), MMP9 (*p*-value = 0.000003) and opticin (*p*-value = 0.0009) was found to be statistically significant thereby indicating an important role of these proteins in ROP pathogenesis (Fig. 1C). Next, we performed validation of MMPs levels in tear samples collected at the time of initial eye screening for preterm born babies. A significantly higher expression of MMP9 was observed in tear samples of the preterm born babies who later progressed to severe ROP as compared to those who developed mild ROP or had regressed disease (Fig. 1D). Since, there was a significant inverse correlation in the opticin levels with MMPs activity (Supplementary Fig. 1), we therefore wanted to assess the type of interactions between the two proteins by in-silico approaches and in-vitro analysis to check if opticin downregulation under hypoxic environment as seen in ROP could be rescued by inhibiting the increased activity of MMPs.

### In-silico analysis

The predicted structure for opticin, and PDB derived structures for pro-MMP (with truncated C-terminal) and active MMP, doxycycline and EDTA are shown in Supplementary Fig. 2. In-silico analysis was done in 2 sets and 4 subsets. First set included protein–protein interaction that included the interactions of pro-MMP9 and active MMP9 separately with opticin respectively (Supplementary Tables S5 and S6). In the second set, to assess if blocking MMPs activity could affect its interaction with opticin, protein–ligand interactions i.e., pro-MMP9 and active MMP9 separately with doxycycline and EDTA were studied.

### Prediction of protein–protein (MMP9 and opticin) interactions

#### Pro MMP9 interacts with opticin

**Figure 2:**
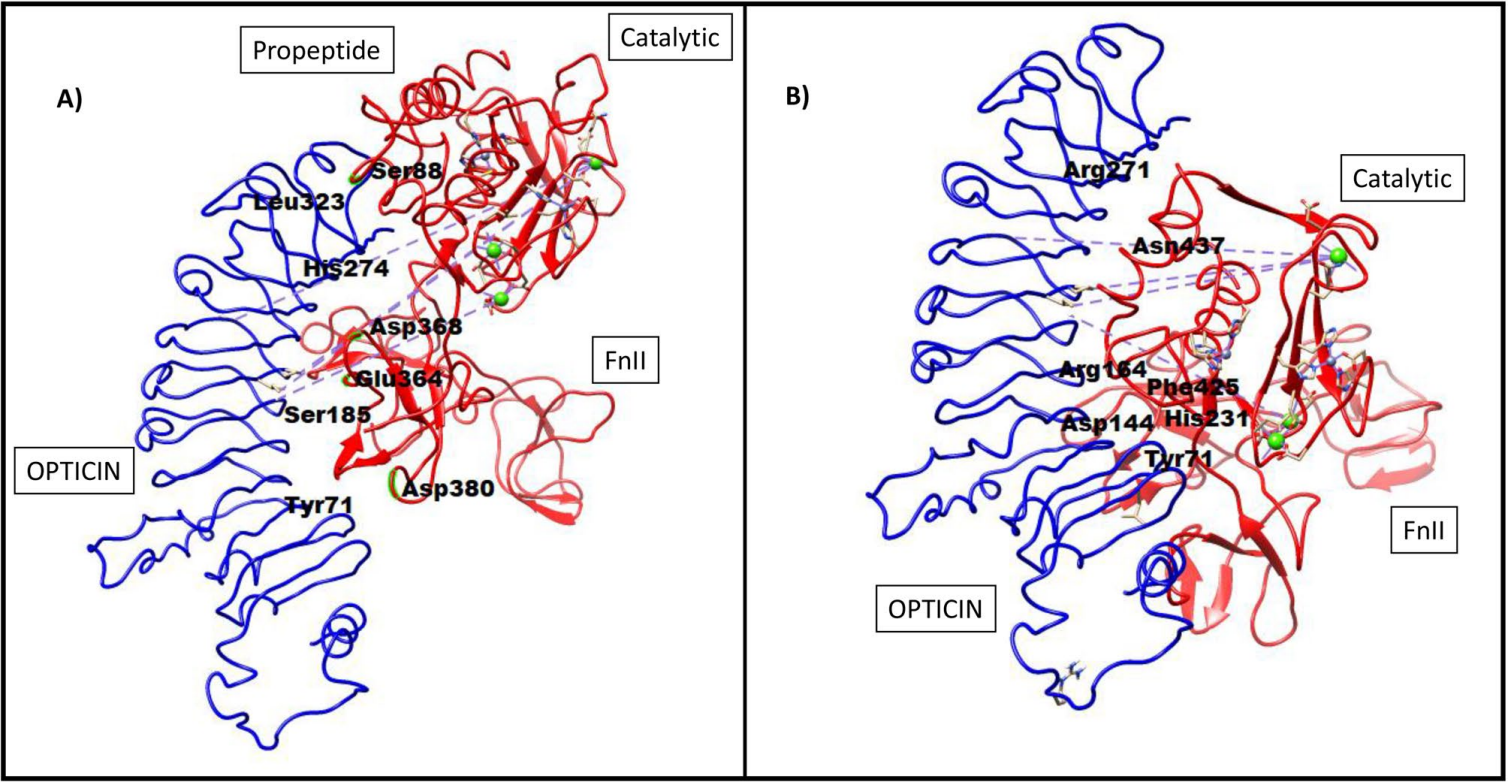
Predicted interactions of opticin with pro-MMP9 (**A**), and active MMP9 (**B**) using PatchDock. The important amino acids involved in these interactions at the predicted sites are shown in bold black colors.

The pro peptide chain of MMP9 interacts with the C terminal domain of opticin. These results also predicted that only FnII domain of pro-MMP9 interacts with opticin. No interactions were seen between pro-MMP9 and the SLRP domain of opticin (Fig. 2A). Predicted interactions among the two proteins included: the interactions of amino acid residue (Asp380, Glu364, Asp368) of FnII domain and amino acid residue (Ser88) of propeptide chain of MMP9 with amino acid (Tyr77, Ser185, His274, Leu323) residues of opticin (Supplementary Table S5).

#### Active MMP9 docked with opticin

On the other hand, for active MMP9, both FnII and catalytic domain (generated after cleaving off the propeptide sequence) interacted with opticin. The catalytic domain of active MMP9 was predicted to interact more strongly with the N-terminal and with amino acids of SLRP domains of opticin protein (Fig. 2B). The predicted interaction between the two proteins included: interactions of amino acid residue (His231, Asn437, His231) of catalytic domain and amino acid residue of FnII domain of active MMP9 with amino acid (Arg164, Arg27, Asp144, Tyr71) residue of opticin (Supplementary Table S6).

### Prediction of protein–ligand (MMP9 and doxycycline) interactions

#### Pro-MMP9: doxycycline interaction

**Figure 3:**
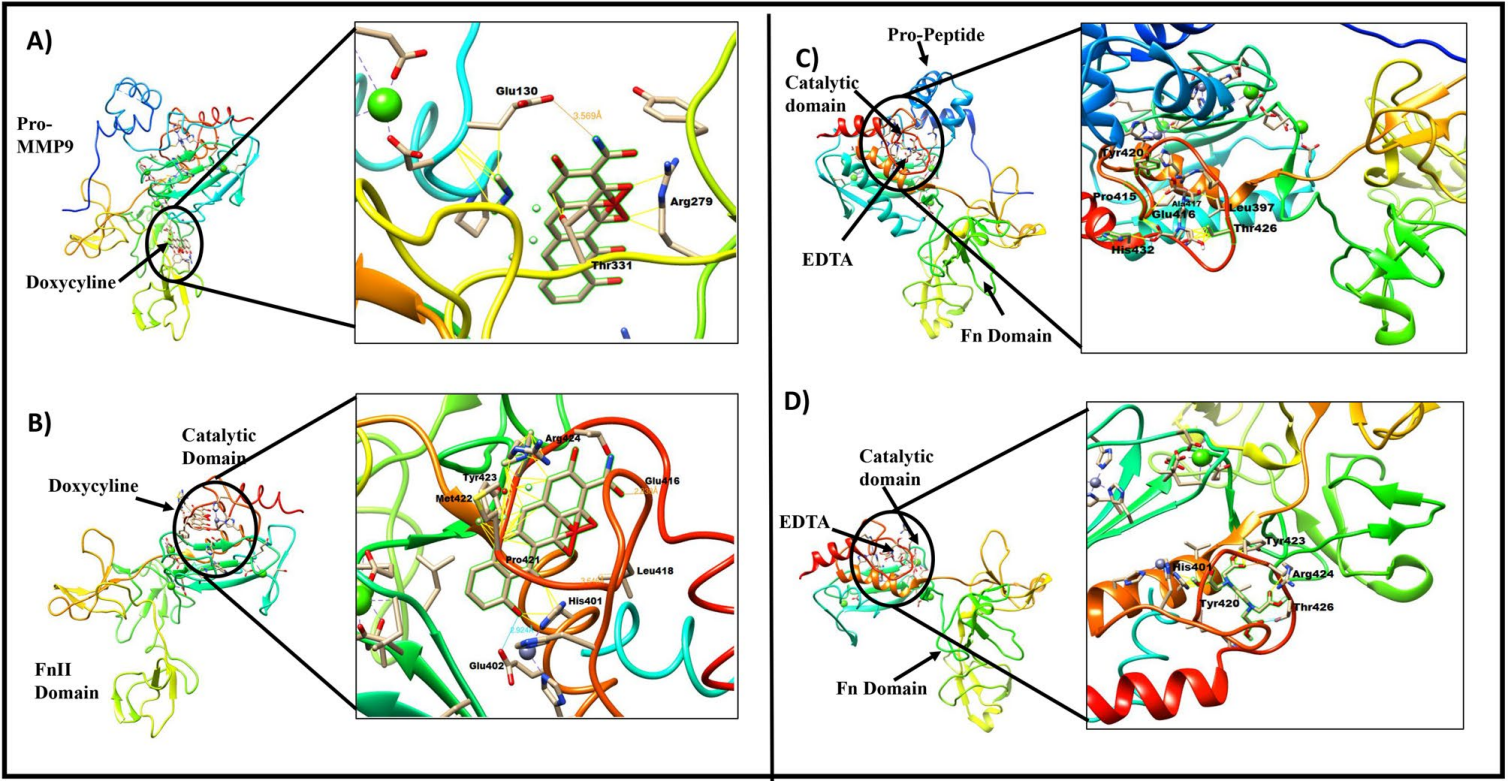
Predicted interactions of doxycycline with pro-MMP9 (**A**), and active MMP9 (**B**). Predicted interactions of EDTA with pro-MMP9 (**C**), and active MMP9 (**D**). The important amino acids involved in these interactions at the predicted sites are shown in bold black colors.

Several different possible interactions were observed between pro-MMP9 and the MMP inhibitor-1 (doxycycline) by PatchDock. (The top 5 results are shown in the Supplementary Table S7). The doxycycline interacts with pro-MMP9 at FnII domain which is required for later’s interaction with opticin (Fig. 3A), a hydrogen bond observed between doxycycline and Glu130 of MMP9 with bond length of 3.569 Å. The other interacting amino acid residues are Thr331, Arg279, and Thr331 (Supplementary Table S7).

#### Active MMP9: doxycycline interaction

Doxycycline interacts with active MMP9 (Fig. 3B) involving its catalytic domain largely by forming hydrogen bonds with amino acid residue (Glu402, Glu416, Leu418) of active MMP9. Besides some other interactions (Arg424, Tyr423, Met422, His401, Pro421) of active MMP9 were also observed. Since, the His401 is already known to interact with zinc ion, the interaction of doxycycline with the catalytic domain (active MMP9) seems to be involved in inhibiting the MMP9 activity (Supplementary Table S8).

#### Pro MMP9: EDTA interaction

Several different possible interactions were observed between pro-MMP9 and MMP inhibitor-2 (EDTA) by PatchDock. (The top 5 results are shown in the Supplementary Table S9). The pro-MMP9 interacts with EDTA at catalytic domain (Fig. 3C), a hydrogen bond observed with amino acid residue (Pro415) and other interactions were observed with amino acid residues (Glu416, Thr426, Ala417, Tyr420, His432) (Supplementary Table S9).

#### Catalytic MMP9: EDTA interaction

EDTA interacts with the catalytic domain of active MMP9 (Fig. 3D) by forming hydrogen bonds with amino acid residue (Arg424, Thr426) of active MMP9 and observed other interactions (Tyr420, Tyr423, His401, Arg424,) with active MMP9 (Supplementary Table S10).

#### Regulation of opticin expression by MMP9 under hypoxic conditions

**Figure 4:**
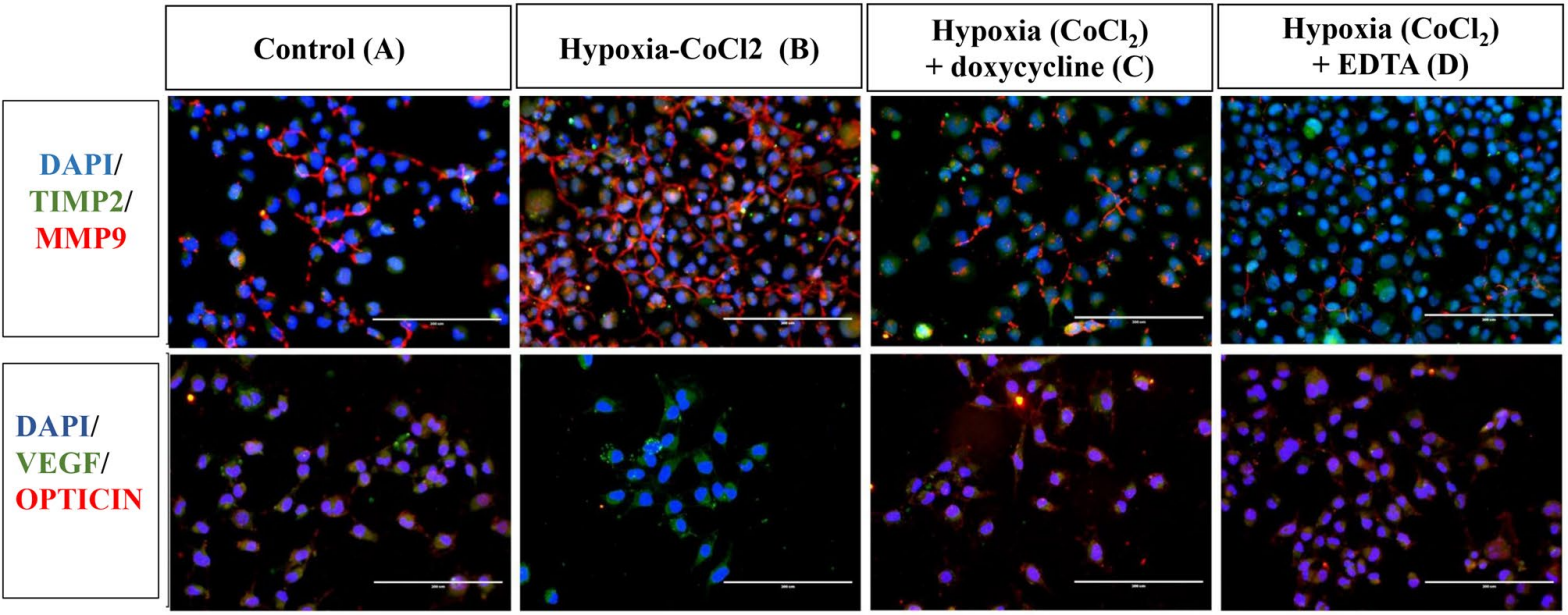
Representative images of immunofluorescence in microglial cells (n = 3, 20× magnified, scale bar 200 μm) showing the expression of MMP9/TIMP2 and VEGF/OPTICIN in control (**A**), hypoxia (CoCl_2_) (**B**), hypoxia (CoCl_2_) + doxycycline (**C**), and hypoxia (CoCl_2_) + EDTA (**D**).

Our in-silico analysis predicted that opticin is acted upon by both pro and active form of MMP9. Next, we assessed the same using cellular assay, if the lower expression of opticin in vitreous of ROP is regulated by increased activity of MMPs in the retina and vitreous. We performed an in-vitro assay in human microglial cells (CHME3 cell line) which revealed a normal expression of the opticin and MMPs in no stress condition. The cells were induced with hypoxic stress by giving a titrated dose of 150 µM cobalt chloride that showed 70% cell survival on alamar blue assay (Supplementary Fig. 3). The hypoxia exposed cells showed higher expression of MMP9 and simultaneous reduced expression of opticin (indicating its degradation). Upon inhibition of MMP9 activity by doxycycline and EDTA the human CHME3 line under hypoxic stress resumed the normal opticin expression while TIMP2 and vascular endothelial growth factor (VEGF) expression remained more or less same (Fig. 4).

#### Relative expression of *MMP9*, *OPTC* and other genes in human CHME3 cell line under hypoxic stress with and without the treatment of specific MMP inhibitors

**Figure 5:**
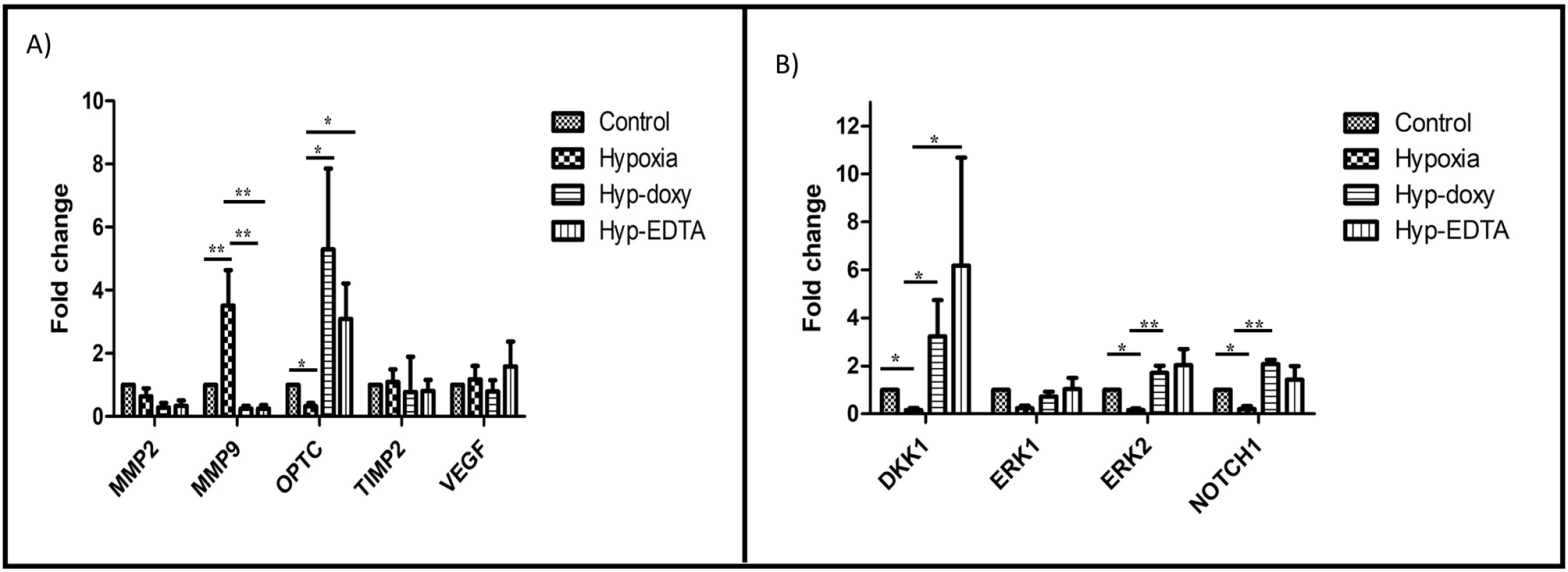
Differential gene expression of *MMP2*, *MMP9*, *OPTC*, *VEGF* and *TIMP2* (**A**), and potential signalling pathway genes (*DKK1*, *ERK1*, *ERK2*, and *NOTCH1*) (**B**) in control, hypoxia (CoCl_2_), hypoxia (CoCl_2_) + doxycycline, and hypoxia (CoCl_2_) + EDTA in microglial cells (n = 3), **p = 0.001, *p = 0.05; data represented as mean ± SEM.

Quantitative real-time PCR based assay for the human microglial cells (CHME3) with and without hypoxic stress also revealed that upon the induction of hypoxia, the microglial cells showed significantly elevated levels of *MMP9* (fold change = 3.52, *p*-value = 0.008**) and a decrease in *OPTC* expression (fold change = 0.32, *p*-value = 0.01*). Further, inhibition of the MMPs activity by doxycycline and EDTA showed significant downregulation of MMPs expression (both doxy; fold change = 0.24, *p*-value = 0.006**, and EDTA; fold change = 0.25, *p*-value = 0.006**) and concurrent upregulation of *OPTC *(both doxy; fold change = 5.2; *p*-value = 0.03* and EDTA; fold change = 3.09; *p*-value = 0.03*) levels. However, not much changes in the expression of *TIMP2* (both doxy; fold change = 0.77; *p*-value = 0.2, EDTA; fold change = 0.81; *p*-value = 0.2) and *VEGF* (doxy; fold change = 0.79; *p*-value = 0.2 and EDTA; fold change = 1.5; *p*-value = 0.4) was seen upon inhibition of MMPs activity under hypoxic condition (Fig. 5A).

#### Exploration of different potential signalling pathways

Induction of hypoxia significantly leads to downregulation of *ERK2* (fold change = 0.16; *p*-value = 0.04*), *DKK1* (fold change = 0.15; *p*-value = 0.03*) and *NOTCH1* (fold change = 0.20; *p*-value = 0.04*), signalling genes but not* ERK1* (fold change = 0.23; *p*-value = 0.08), as compared to controls. Upon inhibition of MMPs activity by doxycycline, all the targeted pathway genes *ERK2* (fold change = 1.71, *p*-value = 0.006**), *DKK1* (fold change = 3.23; *p*-value = 0.02*), and *NOTCH1 *(fold change = 2.07; *p*-value = 0.02*) showed some rescue in expression levels while* ERK1 *levels (fold change = 0.71; *p*-value = 0.14) remained low as of hypoxia treated cells. In the presence of MMP inhibitor 2 (EDTA), the hypoxia treated cells exhibited increased levels of *ERK2* (fold change = 2.02; *p*-value = 0.07) and *DKK1 (*fold change = 6.17; *p*-value = 0.04*) expression but *NOTCH1* (fold change = 1.42; *p*-value = 0.4), and *ERK1* (fold change = 1.03; *p*-value = 0.6), levels approximately remained the same as of control (Fig. 5B).

#### Validation of microglial mediated MMP9 activity in human tissues by the immuno-histochemical examination in fibrovascular membranes of ROP patients

**Figure 6:**
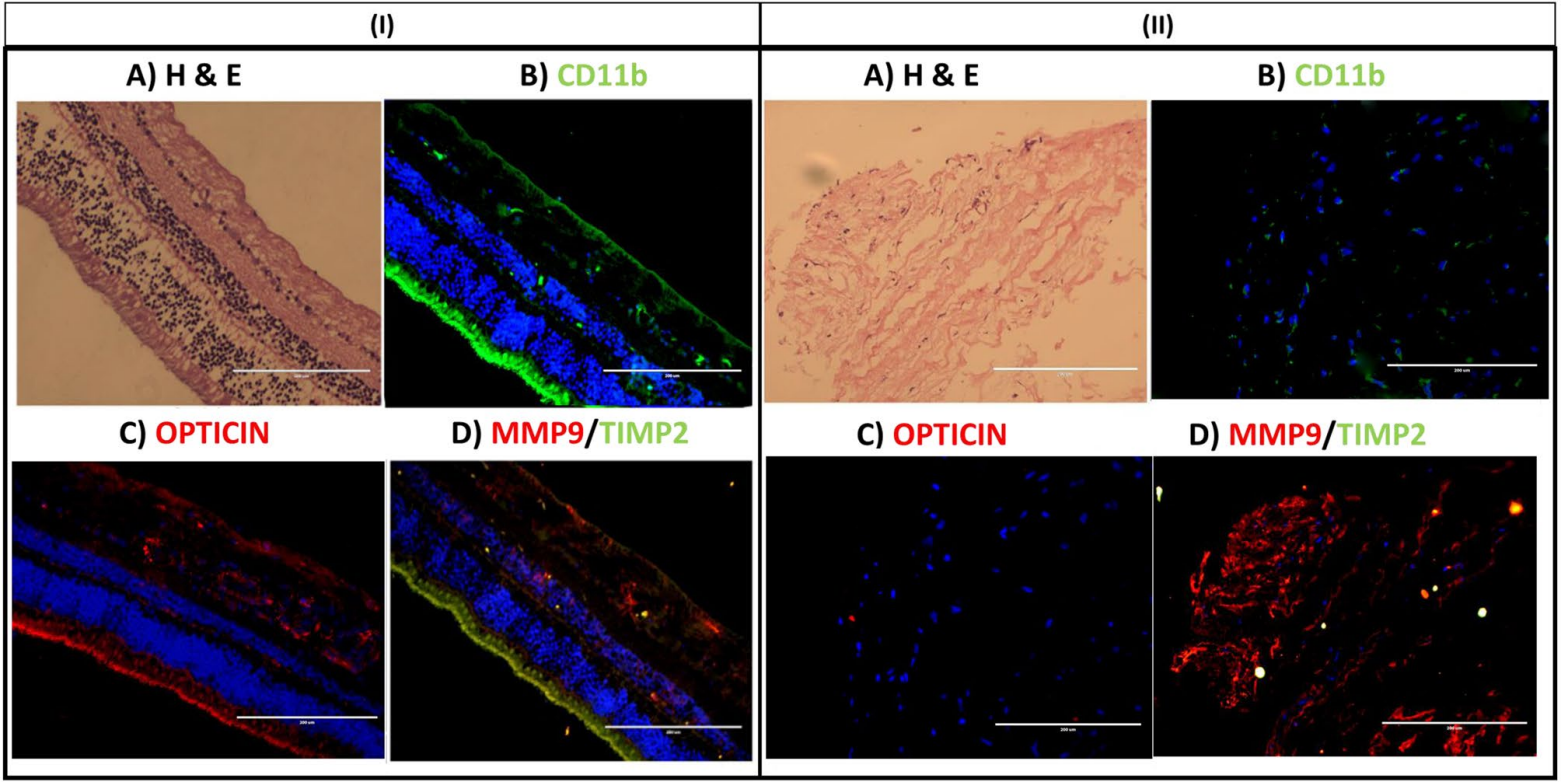
Representative image of H&E (**A**), immunofluorescence of CD11b (**B**), opticin (**C**), MMP9 and TIMP2 (**D**) in normal retinal tissue (I) and fibrovascular membrane (II, n = 3) collected from stage V ROP, (n = 3, 20× magnified, scale bar 200 μm).

In normal retina, microglia (CD11b), TIMP2 and opticin expressions were found in the outer segment (OS), inner nuclear layer (IL), inner plexiform layer (IPL) and in nerve fiber layer (NFL). While MMP9 and opticin was found to be expressed in all retinal layers except photoreceptor layers (Fig. 6. I), in case of ROP fibrovascular membrane, we found higher expression of MMP9 in microglial cells but no TIMP2 and opticin (Fig. 6. II).

## Discussion

ROP is a multifactorial disease leading to childhood blindness worldwide. The disease pathogenesis is poorly understood owing to its complex etiology and access to biological material for research purposes. Activated microglia in the vitreous of ROP patients have been shown to release increased levels of C3 and MMPs in the vitreous^10^. Correspondingly, vitreous mass spectrophotometry results showed that a greater number of opticin peptides are present in the control groups when compared to the ROP probands, indicating opticin levels are dysregulated in ROP disease condition (Our unpublished data). Opticin is an ECM protein with anti-angiogenic properties. Le Goff *et al*. (2012) reported that opticin inhibits the angiogenesis via causing weak adhesions of endothelial cells to collagen mediated by its binding to collagen 1 and 2 and inhibiting collagen’s binding to integrins^15^. The same group further showed that opticin inhibits preretinal neovascularization in OIR model^16^. Ma *et al*. (2012) showed that opticin levels are downregulated under hypoxia via MMP2 in retinal pigment epithelium (RPE) cells^17^. While there are reports on opticin’s role in inhibiting angiogenesis in cellular and animal studies, till date there are no reports available assessing the role of opticin in human ROP cases. In this article, for the first time we focused on MMP9 mediated opticin degradation in the microglia cells. Our results validated reduced opticin levels in the vitreous of ROP patients. Interestingly, there was a strong inverse correlation between the levels of MMP9 and opticin protein in the vitreous humor samples of ROP patients (Supplementary Fig. 1). A study by Tio *et al*. (2014) that found opticin act as substrate for several MMPs resulting in its proteolytic degradation^18^. Thus, we hypothesized that elevated levels of MMPs might degrade the opticin and disturb the homeostatic balance of anti-angiogenic vs angiogenic factors in the retina and vitreous which has been shown to promote preretinal vascularization in oxygen induced retinopathy (OIR) model^16^.

We performed an in-silico analysis that was primarily focused on studying the interactions between MMP9 and opticin to further understand the plausible ways by which MMP inhibition could rescue opticin degradation. Our in-silico analysis also confirmed the possible interaction between MMP9 and opticin and how this could be potentially affected by inhibiting the activity of MMP9. Elkins *et al.* (2002) described the structure of C-terminally truncated MMP9 that has its propeptide attached to the catalytic domain^19^. Since the full-length structure of pro-MMP9 is not available so far, we have used the same structure in the present study to check its interaction with other proteins/ligands^20^. MMP9 is activated by proteolytic cleavage of propeptide leaving catalytic domain available for possible interactions with other proteins. The docking results for doxycycline interactions with pro-MMP9 predicted that doxycycline interact with FnII domain of pro-MMP9 and not with propeptide which predicts that doxycycline does not hinders the conversion of pro-MMP9 to active MMP9, however, this needs to be confirmed by specific protein assays. Further*,* docking of doxycycline with active MMP9, (where the propeptide sequence have been removed) showed the doxycycline’s interactions with His401 which is located in the conserved consensus sequence HExxHxxGxxH of MMP9^19^. His401 interacts with Zn ion^19^, therefore, doxycycline’s interaction with His401, would affect the MMP activity by blocking the Zn ion. Further, the In-silico analysis clearly demonstrated that the catalytic domain of active MMP9 interacts with opticin causing its possible proteolytic degradation. Thus, inhibition of MMPs activity by doxycycline could serve as an effective potential therapy for ROP that needs further detailed investigations in appropriate cells/animal models.

**Figure 7:**
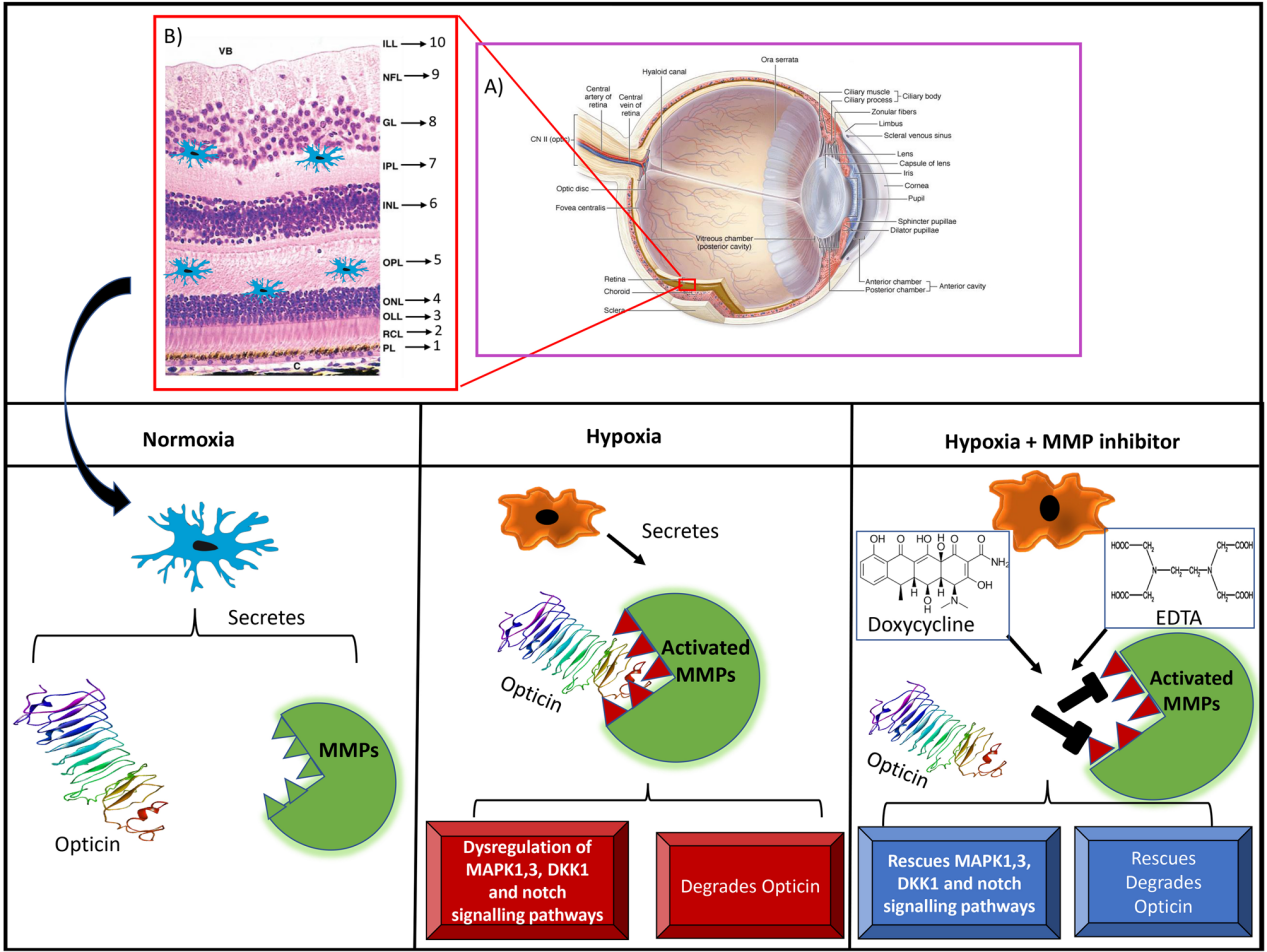
A schematic summary of the role of MMP mediated opticin degradation in microglia under hypoxic stress and in retinopathy of prematurity. Modified and adapted from A: https://quizlet.com/232012123/anatomy-of-the-internal-eye-diagram^39^ B: Jacob Cook (2016)^40^

While an earlier study focused on studying the expression of opticin in RPE cell, in-vitro, we selected the human microglia cell line for studying the microglial cell mediated MMPs activity/inhibition on opticin. Our study for the first time confirmed opticin expression by microglial cells. This was further confirmed on immunohistochemistry of normal retina, showing simultaneous presence of activated microglia (CD11b), TIMP2 and opticin in the outer segment, inner nuclear layer, inner plexiform layer and in nerve fiber layers with MMP9 being expressed in all over the retinal layers. Again, in fibrovascular membrane those are surgically removed from the eyes of severe ROP to prevent retinal detachment, there was a higher expression of MMP9 in microglial positive cells but no opticin. This absence of opticin could be attributed to its degradation by the increased activity of MMPs (Fig. 7). Since fibrovascular membranes are formed under high inflammatory conditions, a further confirmation of this could be attempted in animal models of ROP/OIR.

Under normal conditions, the human microglial cells showed a good expression of opticin, VEGF, TIMP2 and MMP. However hypoxia caused an abnormally high expression of MMP9 and a parallel reduction of opticin levels. This increase in MMP9 activity could be successfully inhibited by EDTA and doxycycline leading to a rescue of opticin reduction caused by its proteolytic degradation by MMPs in microglia. Thus, it clearly demonstrates that not only MMPs are the key regulators of opticin expression in retina but also that MMP inhibition could serve as a possible treatment for checking ROP progression in highly inflamed eyes of preterm babies as confirmed by the tear levels. Doxycycline is an FDA approved drug for the treatment of inflammatory disorder that acts by inhibiting MMP activity. MMPs participate in multiple biological processes via Zinc-dependent multidomain endopeptidases. One of the ways that doxycycline displays its anti-inflammatory properties is by down regulating the transcription/synthesis of MMP9 (stromelysin)^21^ and by selectively chelating the divalent or trivalent metal ion such as Calcium/Magnesium/Zinc. It also inhibits activity of other MMPs and thereby cell division. Hence, further studies on ROP treatment can assess it as a preventive drug to check inflammation in the early stages of the disease after doing clinical trials on suitable animal models of ROP.

Normal expression of MAPK (ERK1/ERK2) is essential for the cell metabolism, differentiation, and proliferation^22^. Flavian *et al*. (2016), showed that doxycycline can inhibit the microglial activation by inhibiting p38 MAPK pathway in primary microglia cells^23^ and thus lowers the proinflammatory cytokines in neurodegenerative conditions. EDTA, the other MMPs inhibitor, is also known to play an important role in most of the receptor mediated signaling mechanisms^24,25^. MMP9 expression can also be induced by the TNF, TGF-β and MAPK/ERK pathways^26–28^. The induction of hypoxia caused a significant increase in the *MMP9* activity in microglial cells and down regulation of *ERK2, DKK1* and *NOTCH1* signaling genes. However, the inhibition of the MMPs activity by doxycycline and EDTA, seemed to rescue the reduction in the major signaling pathway genes that contribute to lower inflammation and cell death. While treatment with doxycycline was able to rescue the expression of targeted pathway genes of *ERK2*, *DKK1* and *NOTCH1* under hypoxia, EDTA could only affect the *ERK2* and *DKK1* expression but not *NOTCH1* and *ERK1.* Additionally, MMPs inhibitors are known to inhibit the NO, ROS and TNF signaling which are essential for the Notch pathway^29–33^. MMPs and DKK (wnt signalling inhibitor) cross talk is required for wnt activation^34^. The results of this study suggest that the inhibition of MMPs by doxycycline and EDTA besides inhibiting MMPs activity that prevents opticin degradation, may also prevent downregulation of *ERK2*, *NOTCH1* and *DKK1* levels under hypoxia. Thus collectively, doxycycline treatment could prevent abnormal proliferation of endothelial cells leading to abnormal angiogenesis in the retina, however, this would need further validation by performing appropriate cellular assays in animal models of ROP.

This study thus, demonstrated that MMPs and other signaling mechanisms are dysregulated under hypoxic stress, which play an important role in ROP pathogenesis and MMP inhibition may be useful for rescuing the expression of opticin and other major signaling genes for the best visual outcome in severe stages of ROP. However the results of this study would require detailed investigation in suitable animal models before this could be use a potential therapy to check progression of ROP.

## Methodology

This study was approved by the Institutional Review Board (IRB) of L.V. Prasad Eye Institute, Hyderabad, India, (LEC02-14-029) and adhered to the tenets of the Declaration of Helsinki. A prior-informed written consent was obtained from the parents/guardians of the study subjects (preterm infants).

### Sample collection and preparation

Tear samples were collected from preterm born babies at the time of their first ocular examination within first month of their birth and then these babies are followed for retinal findings in the subsequent visits. The vitreous humor (50–100 µl) was collected from stage IV and V ROP infants and controls (infants under the age of 1 year with congenital cataract) at the time of pars plana vitrectomy done as part of the routine management of the condition. Both ROP (n = 30) and controls (n = 30) vitreous samples were subjected to RIPA buffer-based lysis followed by acetone precipitation to remove the salt traces and other impurities. The precipitated vitreous proteins were pelleted down at maximum speed for 30 min and dissolved in phosphate buffered saline (PBS). The obtained protein was quantified by using BCA method and normalized to 15 µg.

### Analysis of MMP activity by gelatin zymography

A further validation of increased MMP levels in ROP vitreous sample as seen in our earlier study was performed in the extended cohort. The protein samples were separated in an 8% polyacrylamide gel containing a specific gelatin substrate (4 mg/ml), that is co-polymerized with the acrylamide under non-reducing conditions. After electrophoresis, the gel is washed with triton® X-100 to remove SDS and subsequently incubated at 37 °C for 16 h in a calcium-containing buffer (activation buffer-0.05 M Tris HCl, pH-7.8, 0.2 M NaCl, 5 mM CaCl_2_, 0.02% Brij 35) followed by staining with coomassie brilliant blue solution. The partially renatured enzymes degrade the gelatin leaving a cleared zone that remains unstained appearing as white band under UV light examination. Demographic details of study subjects used for tear samples for zymography showed in the Supplementary Table S2.

### Western blotting

The vitreous samples (10 µg) were also subjected for western blotting for the detection of opticin levels in ROP and no ROP babies. 10% SDS PAGE gel was prepared for the separation of proteins. Pre-stained protein ladder (Cat no# LI-COR, P/N 928-40000) was used for protein sizing. Separated proteins were transferred to a prewet (methanol) PVDF membrane (Cat no# LI-COR, P/N 926-31098). Ponceau staining was done for blots to confirm equal loading and complete transfer of proteins from gel to membrane in each lane before performing blocking for an hour with Odyssey blocking buffer (Cat no# LI-COR, P/N 927-40000). The blot was incubated with rabbit polyclonal opticin antibody (ab170886, abcam) (1:500) overnight. The blot was washed with PBST thrice and stained with anti-rabbit fluorescence labeled secondary antibody (LI-COR, Cat no LI-COR, P/N 928-40006). The blot was washed thrice with PBST followed by 1 × PBS to remove any unbound secondary antibody. The signals were detected by Infra-red (IR) based imager (Odyssey, LI-COR, USA). MMP2, MMP9 (zymography) and opticin (western blotting) band intensities were measured by using Image J, A correlation analysis for total MMPs-opticin and MMP9-opticin levels was performed in a subset of cases and controls (Supplementary Fig. 1). Demographic details of study subjects used for vitreous western blotting analysis showed in the Supplementary Table S1.

### In-silico analysis for protein–protein and protein: ligand interactions

Protein–protein (MMP9-opticin) interaction and protein–ligand (MMP9-doxycycline and MMP9-EDTA) interactions were studied by in-silico analysis to understand how MMPs degrades opticin in the eye and further how inhibiting the MMP activity using specific MMP inhibitors rescues the opticin levels. Since it was not very clear from the existing literature that whether doxycycline chelates MMP9 in its pro or active form and its interaction with opticin, we performed both protein–protein (MMP9-opticin) and protein–ligand (MMP9-doxycycline) docking.

The protein structure of pro-MMP9 protein (Protein Data Bank PDB id: 1L6J) and the ligand doxycycline (DB00254) and EDTA (DB00974) were retrieved from protein data-bank and drug bank respectively (Supplementary Fig. 2). Active MMP9 was obtained from pro-MMP9 by deleting out the propeptide sequence. Since opticin structure was not available, therefore its structure prediction was done by the threading method after submitting the protein FASTA sequence to I-TASSER (https://zhanglab.ccmb.med.umich.edu/I-TASSER/). The best predicted structures were then selected based on C scores, RMSD score and TM score^35,36^.

The protein PDB and ligand PDB structures were uploaded to PatchDock server^37,38^ for protein–protein and protein–ligand docking respectively (https://bioinfo3d.cs.tau.ac.il/PatchDock/) with the clustering RMSD of 4 A and complex type as default value was provided to the server.

### Human microglial cell culture for studying the activity of MMPs under hypoxic condition

The human microglial cell line (CHME3, n = 3) was cultured in DMEM medium containing 10% FBS along with 1% antibiotics (penicillin and streptomycin) and then exposed to different conditions to check the activity of MMPs on different proteins. For these experiments, approximately 10,000 cells per well were used. Alamar blue assay was done to observe the cytotoxic effect (Supplementary Fig. 3) and determining the optimum concentration of CoCl_2,_ EDTA and doxycycline for the subsequent experiments (Life technologies, Cat.no DAL1025). Serum depleted for 6 h followed by exposure to hypoxia by treating with cobalt chloride (150 µM) and with MMP inhibitors (EDTA-10 µg, doxycycline-20 µg) for 24 h, untreated cells were used as control.

### Immunofluorescence

After 24 h treatment, microglia cells (n = 3) were washed with 1 × PBS and fixed with 4% formaldehyde for 10 min at room temperature followed by 3 washes with 1 × PBS. The cells were subjected to 0.3% of triton X 100 treatment at RT for 10 min for permeabilization followed by blocking using 2% BSA (HIGH MEDIA, Cat.no TC194) for 1-h. Cells were incubated overnight at 4 °C in appropriate dilution of primary antibody (MMP9*,* opticin*,* VEGF*,* and TIMP2). The antibody details for the proteins analyzed are provided in Supplementary Table S3. To remove unbound primary antibody, the cells were washed thrice with 1 × PBS. Fluorescent labeled secondary antibodies were used for the detection and then mounted with slow fade gold antifade containing DAPI (Life technologies, ref. S36939). Expression of targeted proteins in microglial cells under different conditions was studied under EVOS fluorescent microscope. A comparative quantitative analysis (Image J analysis) of signal intensities for MMPs and opticin expression in the microglia cells was performed in different categories (Supplementary Fig. 4).

### Relative gene expression quantification

RNA was extracted from the same set of the human microglial cells (n = 3) by Trizol method. Quality and quantity of extracted RNA was measured by nanodrop and gel electrophoresis. 500 ng of final concentration of RNA was used for the cDNA conversion using thermostatic verso cDNA synthesis kit (AB1453B). Expressions of *MMP9, OPTC, VEGF* and *TIMP2* were assessed by qPCR using SYBR green chemistry (Biorad cat.no 38220090).

Various potential signaling pathways involved in angiogenesis and ECM reorganization (*ERK1, ERK2, NOTCH1, and DKK1)* that might have been affected by alterations in *MMP*s and *OPTC* levels, were assessed under normal and hypoxic conditions, with and without the treatment of MMP inhibitors (doxycycline and EDTA) treatments. Expressions of *ERK1, ERK2, NOTCH1, and DKK1* were assessed by qPCR. The primer details for the genes analyzed are provided in Supplementary Table S4.

### Immunohistochemistry

The changes in MMPs and opticin levels under normal and hypoxic stress in the human microglial cells were further correlated in diseases tissue by performing IHC on fibrovascular membrane obtained from ROP patients (n = 3) along with normal cadaveric retina (n = 1) as a positive control. The fibrovascular membranes formed at vitreo-retinal junctions are removed as a part of routine surgery (membrane peeling) and immediately frozen the tissue in OCT tissue freezing medium in the tissue mold. 5 μm thick sections were taken on charged slides. To check the orientation and quality of tissue hematoxylin and eosin staining was performed. The slides were washed 3 times with 1 × PBS. To permeabilize, 0.3% of triton X 100 treatment was given for 10 min followed by blocking with 2% BSA for 1-h. Incubated the fibrovascular membrane overnight at 4 °C in appropriate antibody (MMP9, opticin, VEGF, and TIMP2) diluted in the 1% BSA (Supplementary Table S3). Fluorescent labeled secondary antibodies were used for the detection. Expression of targeted protein in fibrovascular membrane was identified by EVOS fluorescent microscope.

## Supplementary Information

Supplementary Information

## Data Availability

The authors declare that [the/all other] data supporting the findings of this study are available within the paper [and its Supplementary information files].

## Abbreviations

ROP: Retinopathy of prematurity
MMP9: Matrix metalloproteinase 9
MMP2: Matrix metalloproteinase 2
OPTC: Opticin
VEGF: Vascular endothelial growth factor
BRB: Blood-retinal barrier
ECM: Extracellular matrix
TIMP1: Tissue inhibitory metalloproteases 1
TIMP2: Tissue inhibitory metalloproteases 2
C3: Complement component 3
SLRPs: Small leucine rich proteins
EDTA: Ethylene diamine tetraacetic acid
dNTPs: Deoxy nucleotide triphosphates
bp: Base pair
IgG: Immunoglobulin G
IHC: Immunohistochemistry
IF: Immunofluorescence
FnII: Fibronectin type II
PDB: Protein data bank
CoCl_2_: Cobalt chloride
ERK1: Extracellular signal-regulated kinases 1
ERK2: Extracellular signal-regulated kinases 2
DKK1: Dickkopf WNT signaling pathway inhibitor 1
NOTCH1: Notch receptor 1
CHME3: Microglia cell line
RPE: Retinal pigment epithelium
EC: Endothelial cells
ml: Millilitre
M: Molar
μl: Microliter
mM: Millimolar
ng: Nanogram
μg: Microgram
μM: Micromolar
GA: Gestational age
BW: Birth weight
OIR: Oxygen induced retinopathy
PAGE: Polyacrylamide gel electrophoresis
NPE: Non-pigmented epithelial
RGH: Retinal growth hormone
OS: Outer segment
IL: Inner nuclear layer
IPL: Inner plexiform layer
NFL: Nerve fiber layer
TNF: Tumor necrosis factor
TGF beta1: Transforming growth factor beta 1
ROS: Reactive oxygen species
NO: Nitric oxide
