# Supplementary material for: An interplay of microglia and matrix metalloproteinase MMP9 under hypoxic stress regulates the opticin expression in retina
Source: Sci Rep. 2021 Apr 2;11:7444. doi: 10.1038/s41598-021-86302-2 (PMC8018966; doi:10.1038/s41598-021-86302-2)
Supplement: Supplementary file 1 — Supplementary Information [file 41598_2021_86302_MOESM1_ESM.docx]

**Supplementary information**

**An interplay of microglia and matrix metalloproteinase MMP9 under hypoxic stress regulates the Opticin expression in retina**

**Authors: Satish Patnaik B^1^*, Meenakshi Rai^1^*, Subhadra Jalali^2^, Komal Agarwal^2^, Akshay Badakere^3^, Lavanya Puppala^1^, Sushma Vishwakarma^1^, Divya Balakrishnan^2^, Padmaja K. Rani^2^, Ramesh Kekunnaya^3^, Preeti Patil Chhablani^3^, Subhabrata Chakrabarti^1^, Inderjeet Kaur^1^***

Affiliation(s):

^1^Prof. Brien Holden Eye Research Centre,

^2^Smt. Kannuri Santhamma Centre for Vitreo Retinal Diseases,

^3^Jasti V Ramanamma Children’s Eye Care Centre, L V Prasad Eye Institute, Hyderabad, India

*These authors contributed equally to this work

**Corresponding Author**

**Inderjeet Kaur**

Prof Brien Holden Eye Research Centre, LV Prasad Eye Institute, Hyderabad, India,

[inderjeet@lvpei.org](mailto:inderjeet@lvpei.org), Tel. no. +91- 40-68102508, Fax no. +91-40-23548271

**SUPPLEMENTARY TABLE S1 |** Demographics details of study subjects used for vitreous western blotting analysis


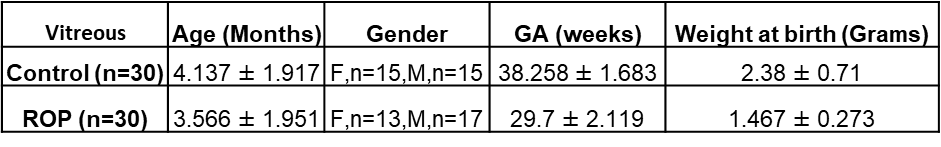


**SUPPLEMENTARY TABLE S2 |** Demographics details of study subjects used for tear samples for zymography

|  | **Mean + SD** | | | |  |
| --- | --- | --- | --- | --- | --- |
| **Tear** | **Age (Months)** | **GA (weeks)** | **Weight at birth (Kg)** | **O_2_ supply (days)** | **Gender** |
| Controls | 3.27 + 2.94 | 25.2+ 10.3 | 1.43+ 0.23 | 5.92 + 2.60 | M:n=8,F,n=10 |
| Mild ROP-regressed | 3.3 + 2.50 | 21.3+ 11.6 | 1.39+ 0.22 | 6.28 + 0.92 | M:n=8,F,n=5 |
| Mild ROP-progressed | 3.58 + 2.64 | 29.3+ 1.2 | 1.26+ 0.18 | 5.25 + 2.05 | M:n=5,F,n=7 |
| Severe ROP | 8.4 + 1.68 | 24.5+ 9.2 | 1.21+ 0.25 | 5.57 + 1.3 | M:n=10,F,n=6 |

(Note- GA-Gestational age, Kg-Kilogram, M-Male, F-Female)

**SUPPLEMENTARY TABLE S3 |** Details of antibodies used for western blotting, Immunofluorescence and immunohistochemistry and their dilutions

| S.No | Antibody | Dilutions(μL) | Company | Catalogue No. | Country |
| --- | --- | --- | --- | --- | --- |
| 1 | Rabbit-Opticin | 1.500 | Abcam | ab170886 | USA |
| 2 | Rabbit-MMP9 | 1.500 | Abcam | ab38898 | USA |
| 3 | Mous-TIMP2 | 1.300 | santacruz | sc-365671 | USA |
| 4 | Mouse-VEGF | 1.200 | R&D | MAB 293 | USA |
| 5 | Goat anti Rb 488 | 1:300 | Life Tech. | A-11008 | USA |
| 6 | Goat anti Ms 48 8 | 1:300 | Life Tech. | A-10680 | USA |
| 7 | Goat anti Ms 594 | 1:300 | Life Tech. | A-11005 | USA |
| 8 | Goat anti Rb594 | 1:300 | Life Tech. | A-11012 | USA |
| 9 | Anti -Rb 800CW  Licor) | -- | 1:10,000 | P/N 928-40006 | USA |

**SUPPLEMENTARY TABLE S4** | Primer sequence and SYBR green based assays used for qRT PCR

| S.no | Gene | Forward primer (5’-3’) | Reverse primer (5’-3’) |
| --- | --- | --- | --- |
| 1 | *OPTC* | AACCGCATCAGCCGTATC | CGGAAGGCATCATTATCG |
| 2 | *MMP9* | FH3_MMP9(Sigma code for forward and reverse primers) | |
| 3 | *TIMP2* | CTTCCACAGGTCCCACAACC | CAGCCCTGGCTCCCGAGGC |
| 4 | *VEGF* | ATCTTCAAGCCATCCTGTGTGC' | CAAGGCCCACAGGGATTTTC |
| 5 | *MAPK1* | CAGGGAAGATGGGCCGGTTAGAGA | TGAAGCGCAGTAAGATTTTT |
| 6 | *MAPK 3* | CCTGCGACCTTAAGATTTG | CAGGGAAGATGGGCCGGTTAGAGA |
| 7 | *NOTCH1* | TTGGGAGGAGCAGATTTTTG | CACTGGCATGACACACAACA |
| 8 | *DKK1* | GATCATAGCACCTTGGATGGG | GGCACAGTCTGATGACCGG |
| 9 | *β Actin* | TCTACAATGAGCTGCGTGTG | GGTGAGGATCTTCATGAGGT |

**SUPPLEMENTARY TABLE S5 |** Top 5 result of pro-MMP9 interacting with opticin

| Solution No. | Score | Area | ACE | Transformation |
| --- | --- | --- | --- | --- |
| 1 | 15816 | 2186.60 | 200.67 | -0.08, -1.17, 1.13, 107.31, 43.07, 115.75 |
| 2 | 15584 | 2894.00 | 28.14 | 2.76, -0.53, 1.39, 20.04, 71.81, 136.40 |
| 3 | 15570 | 2272.00 | 465.39 | -1.28, -0.61, 3.12, 75.40, 129.90, 146.40 |
| 4 | 15498 | 3286.80 | 320.23 | -0.41, -1.07, 1.93, 146.67, 59.36, 116.14 |
| 5 | 15342 | 2857.50 | 289.39 | -0.41, -0.98, 1.77, 144.96, 49.54, 114.19 |

**SUPPLEMENTARY TABLE S6 |** Top 5 result of active-MMP9 interacting with opticin

| Solution No. | Score | Area | ACE | Transformation |
| --- | --- | --- | --- | --- |
| 1 | 15834 | 2455.30 | 345.88 | -0.04, -0.98, 2.08, 152.52, 50.70, 100.23 |
| 2 | 15170 | 2253.40 | 440.23 | 0.87, -0.75, -3.01, 140.56, 37.11, 64.46 |
| 3 | 14566 | 1897.10 | 246.66 | -0.93, -0.46, -2.74, 96.59, 166.37, 97.51 |
| 4 | 14520 | 2272.80 | 289.83 | -0.57, 0.46, -0.56, 10.63, 30.76, 63.92 |
| 5 | 14508 | 1973.20 | 458.89 | 1.99, 0.35, 0.93, 48.40, 76.66, 50.74 |

**SUPPLEMENTARY TABLE S7 |** Top 5 result of doxycycline interacting with pro-MMP9

| Solution No. | Score | Area | ACE | Transformation |
| --- | --- | --- | --- | --- |
| 1 | 5136 | 610.30 | -138.98 | -2.91, -0.02, 3.08, 72.84, 40.28, 31.58 |
| 2 | 5076 | 688.00 | -228.27 | -2.03, 0.74, 1.44, 55.13, 28.39, 0.84 |
| 3 | 5050 | 621.80 | -239.31 | -2.46, 1.17, 0.89, 34.41, 41.63, -4.83 |
| 4 | 5006 | 608.10 | -245.66 | 1.89, -1.01, -2.79, 24.58, 58.75, 65.52 |
| 5 | 4938 | 573.30 | -185.54 | 1.70, 0.97, 3.03, 59.40, 51.49, 14.32 |

**SUPPLEMENTARY TABLE S8|** Top 5 result of interacting doxycycline with active MMP9

| Solution No. | Score | Area | ACE | Transformation |
| --- | --- | --- | --- | --- |
| 1 | 5288 | 700.00 | -410.38 | -1.18, -0.24, 0.37, 4.55, 46.42, 29.74 |
| 2 | 5226 | 646.30 | -337.58 | 2.68,1.16, 2.04, 63.72, 27.09, 29.00 |
| 3 | 5072 | 607.30 | -344.32 | -2.59, -1.07, -0.86, 13.53, 45.26, 77.19 |
| 4 | 5018 | 594.20 | -375.56 | -1.24, -0.39, -2.70, 74.02, 46.57, 49.84 |
| 5 | 4958 | 565.30 | -290.42 | 1.75, -0.37, -2.89, 58.59, 43.72, 79.18 |

**SUPPLEMENTARY TABLE S9 |** Top 5 result of EDTA interacting with pro-MMP9

| Solution No. | Score | Area | ACE | Transformation |
| --- | --- | --- | --- | --- |
| 1 | 3882 | 442.10 | -263.60 | -0.44, 0.10, -2.90, 44.95, 53.38, 39.67 |
| 2 | 3694 | 469.20 | -292.29 | 1.70, -0.11, 0.53, 42.31, 51.92, 39.31 |
| 3 | 3682 | 437.80 | -226.96 | 0.02, 0.22, 0.22, 45.57, 53.48, 39.56 |
| 4 | 3672 | 470.90 | -279.97 | -1.42, -0.16, 0.64, 42.23, 52.77, 39.89 |
| 5 | 3674 | 382.60 | -88.28 | -2.74, -1.17, -1.89, 25.69, 51.93, 32.75 |

**SUPPLEMENTARY TABLE S10 |** Top 5 result of EDTA interacting with active-MMP9

| Solution No. | Score | Area | ACE | Transformation |
| --- | --- | --- | --- | --- |
| 1 | 3860 | 477.60 | -278.82 | 1.57, 0.22, -2.70, 41.98, 52.42, 39.33 |
| 2 | 3746 | 465.10 | -270.40 | 0.79, -0.13, -0.08, 44.36, 52.88, 38.99 |
| 3 | 3678 | 433.70 | -263.71 | -0.44, 0.22, -2.96, 45.13, 52.98, 39.62 |
| 4 | 3672 | 470.60 | -278.26 | -1.42, -0.16, 0.64, 42.23, 52.77, 39.89 |
| 5 | 3598 | 415.80 | -203.26 | -3.08, 0.08, 0.12, 46.61, 54.07, 39.07 |


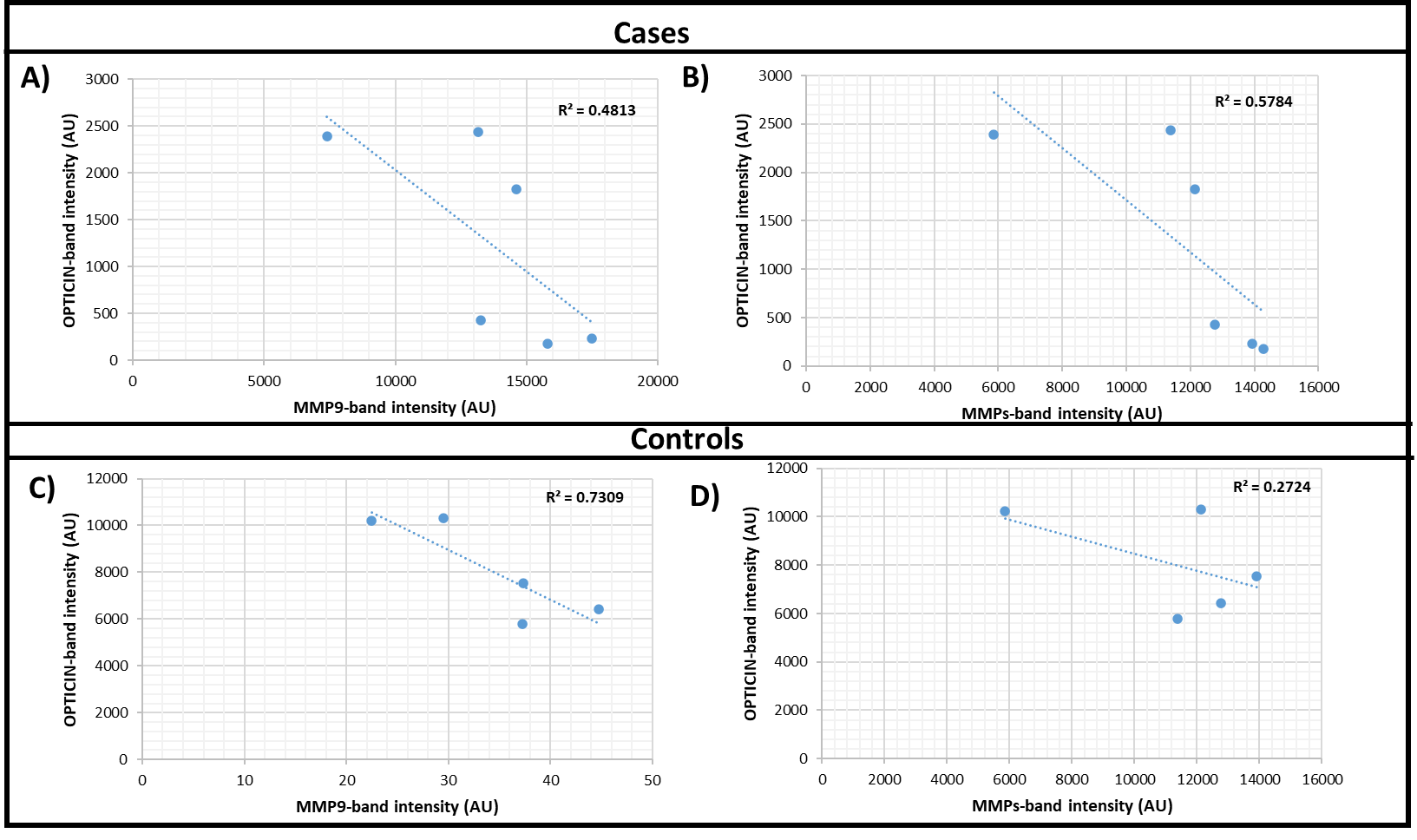


**SUPPLEMENTARY FIGURE 1**| Correlation graphs shows negative correlation between MMPs and opticin in cases-MMP9 (A), MMPs (B), controls-MMP9 (C) and MMPs (D).


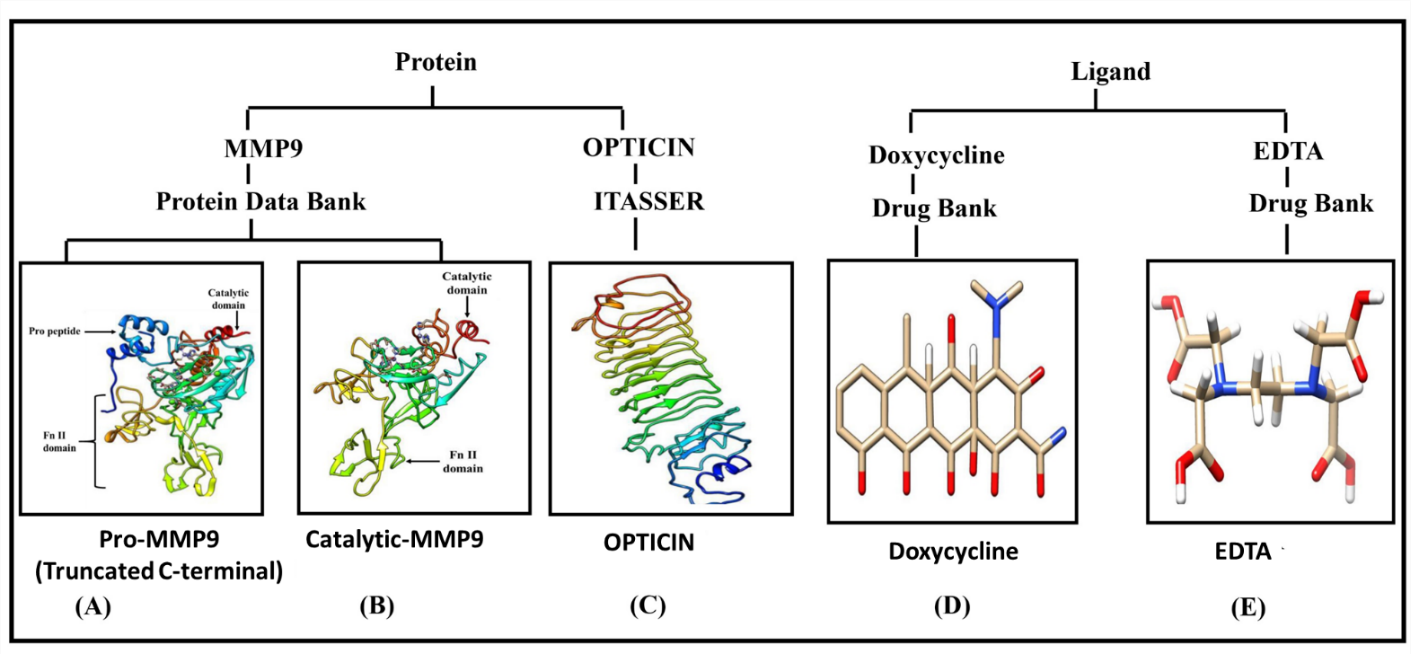
 **SUPPLEMENTARY FIGURE 2 |** Representative images of (A) structure of pro-MMP9 (C-terminally truncated pro-MMP9 retrieved from protein data bank-PDB ID: 1L6J), consist of propeptide (residues 20-109), catalytic domain (residues 110-215 and 390-444) and the 3 FnII domains (residues 216-389)^22^ (B) Catalytic MMP9 is obtained from pro-MMP9 by deleting propeptide residues 20-109 using VMD tool. Catalytic MMP9 comprises of the catalytic domain and 3 FnII domain (C) Opticin structure is generated by threading method using I-TASSER (structure is not available in PDB database) (D) Structure of doxycycline (DB00254), and (E) EDTA (DB00974) obtained from drug bank


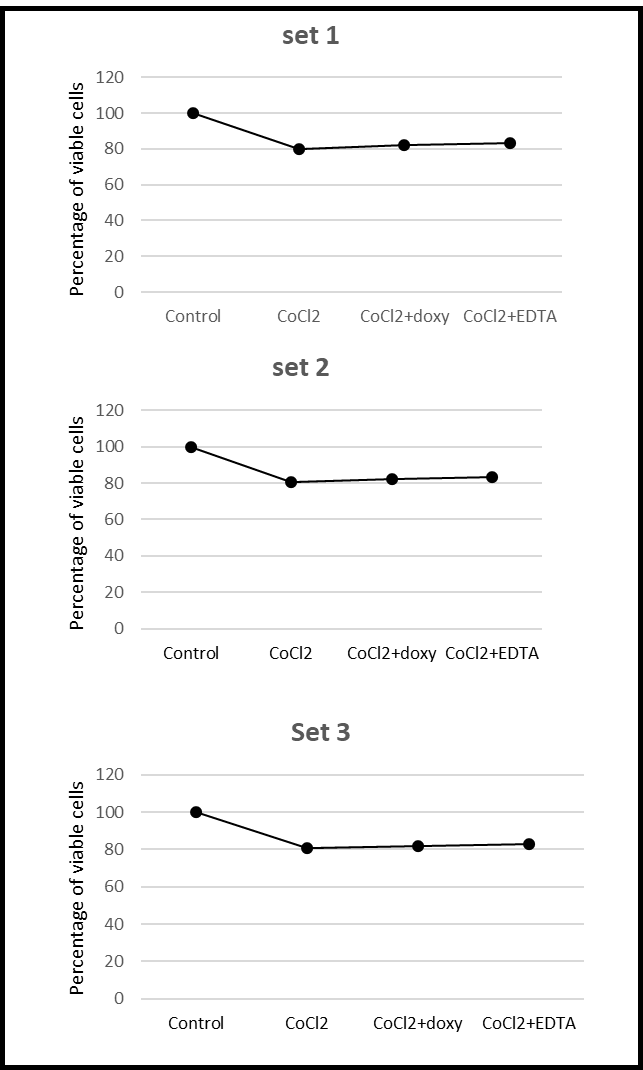


**SUPPLEMENTARY FIGURE 3 |** The microglia cell lines were treated with CoCl2, CoCl2 with doxycycline and CoCl2 with EDTA for a period of 24 h. The viability was measured using Alamar blue–based dose-dependent cell viability assay (N = 3 biological and technical replicates).


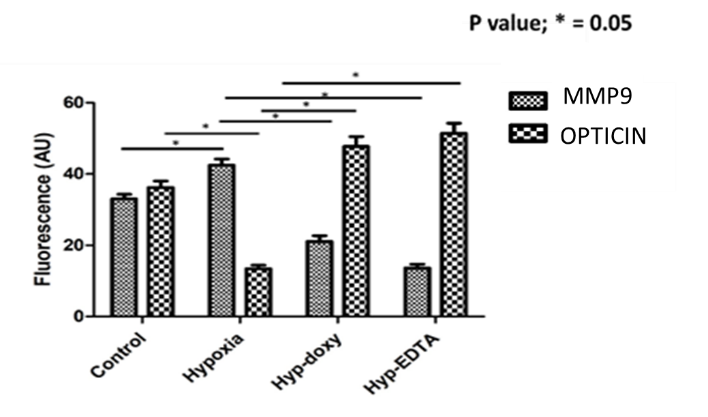


**SUPPLEMENTARY FIGURE 4** | Differential expression of opticin and MMP9 in control, hypoxia (CoCl_2_), hypoxia (CoCl_2_) + doxycycline, and hypoxia (CoCl_2_) + EDTA in microglial cells (n=3), *p = 0.05; data represented as cells fluorescence expression mean ± SEM.
